# Scombroid Fish Poisoning

**DOI:** 10.4269/ajtmh.21-1345

**Published:** 2022-03-21

**Authors:** Walter A. Eyer-Silva, Vivian Paola Arteaga Hoyos, Livia Nascimento

**Affiliations:** Hospital Universitário Gaffrée e Guinle, Centro de Ciências Biológicas e da Saúde, Universidade Federal do Estado do Rio de Janeiro (UNIRIO), Rio de Janeiro, Brazil

**Figure 1.**
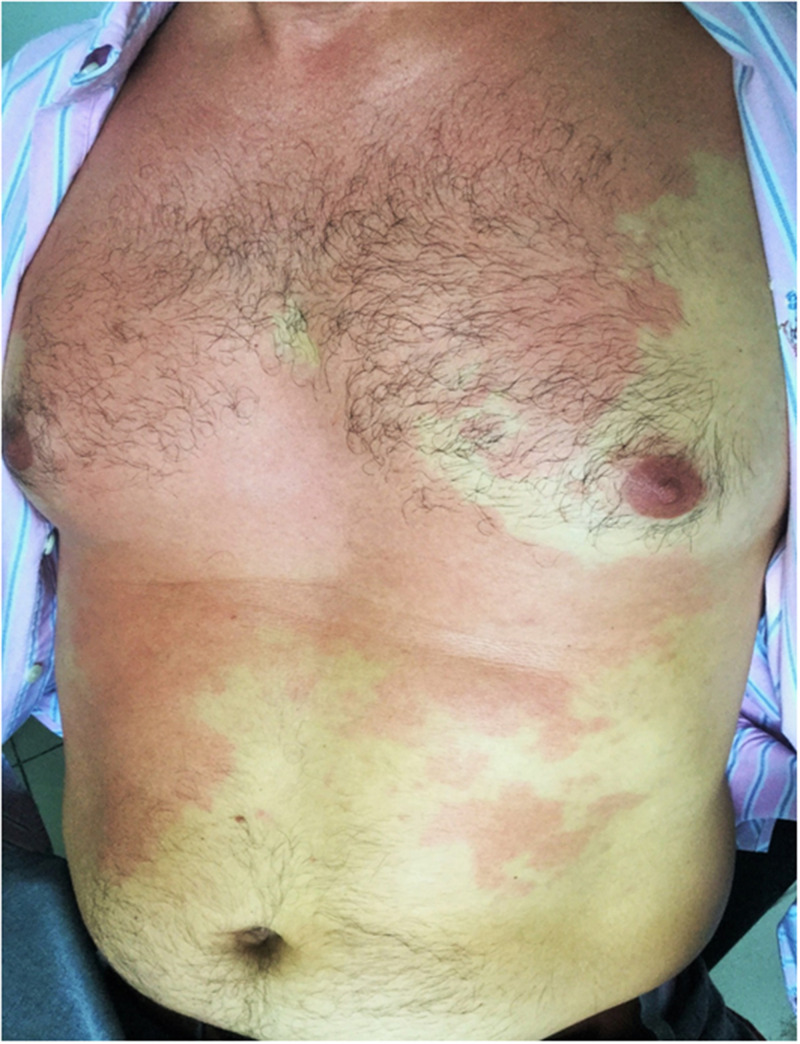
Large areas of flushing without wheals over the trunk of a 42-year-old man. The rash was first noted 10 minutes after consumption of tuna fish. This figure appears in color at www.ajtmh.org.

A 42-year-old man presented with a 60-minute history of a burning and itching cutaneous eruption, accompanied by headache and metallic dysgeusia. Large areas of flushing without wheals, mainly over the face and trunk, were noted (Figure 1). Vital signs were normal. The rash was first noted 10 minutes after the consumption of tuna fish (*Thunnus* spp.) in a restaurant near Rio de Janeiro’s financial district. He had no history of fish allergy or other food or drug hypersensitivity reactions. An antihistamine was prescribed, with complete resolution within 24 hours. He developed no hypersensitivity reactions on subsequent ingestions of tuna. The clinical picture was considered consistent with ichthyosarcotoxism resulting from scombroid fish poisoning.

Several marine animals are capable of producing toxins that can induce seafood-related disease.^1^ Ichthyosarcotoxism refers to seafood-related disease caused by the ingestion of toxins present in fish tissues. Histamine fish poisoning, also known as scombroid fish poisoning, is a foodborne illness associated with the consumption of fish that has been stored improperly after being caught.^2^ The term scombroidism derives from the dark-meat species of the Scombridae family (such as tuna, bonito, skipjack, marlin, and mackerel), although many other non-scombroid species (such as herring, sardines, anchovies, and mahi-mahi) may be implicated in the syndrome.^3^ The condition is probably underreported, underrecognized, and misdiagnosed as an IgE-mediated fish allergy. Presentation shares a resemblance to an allergic reaction and varies from a mild, short, self-limited illness to severe and life-threatening poisoning.^3^^–^^5^

These fish share in common high levels of free histidine in their muscle tissues. If not kept at a temperature of ≤ 0°C immediately after being caught, Gram-negative bacteria present in fish gills and gastrointestinal tract microbiota convert histidine into histamine through the activity of histidine decarboxylase. Once formed, histamine is resistant to cooking, smoking, freezing, and canning.^4^ Potentiation of histamine toxicity by other compounds present in spoiled fish has also been suggested.^5^ Immediate refrigeration after fishing will prevent histamine production.
